# Biomechanical evaluation of multiple pelvic screws and multirod construct for the augmentation of lumbosacral junction in long spinal fusion surgery

**DOI:** 10.3389/fbioe.2023.1148342

**Published:** 2023-03-14

**Authors:** Honghao Yang, Aixing Pan, Yong Hai, Fengqi Cheng, Hongtao Ding, Yuzeng Liu

**Affiliations:** Department of Orthopedic Surgery, Beijing Chao-Yang Hospital, Beijing, China

**Keywords:** sacropelvic fixation, multiple screw, multirod construct, lumbosacral junction, finite element, Biomechanics, spinal fusion, spinal deformity

## Abstract

**Background:** Posterior long spinal fusion was the common procedure for adult spinal deformity (ASD). Although the application of sacropelvic fixation (SPF), the incidence of pseudoarthrosis and implant failure is still high in long spinal fusion extending to lumbosacral junction (LSJ). To address these mechanical complications, advanced SPF technique by multiple pelvic screws or multirod construct has been recommended. This was the first study to compare the biomechanical performance of combining multiple pelvic screws and multirod construct to other advanced SPF constructs for the augmentation of LSJ in long spinal fusion surgery through finite element (FE) analysis.

**Methods:** An intact lumbopelvic FE model based on computed tomography images of a healthy adult male volunteer was constructed and validated. The intact model was modified to develop five instrumented models, all of which had bilateral pedicle screw (PS) fixation from L1 to S1 with posterior lumbar interbody fusion and different SPF constructs, including No-SPF, bilateral single S2-alar-iliac (S2AI) screw and single rod (SS-SR), bilateral multiple S2AI screws and single rod (MS-SR), bilateral single S2AI screw and multiple rods (SS-MR), and bilateral multiple S2AI screws and multiple rods (MS-MR). The range of motion (ROM) and stress on instrumentation, cages, sacrum, and S1 superior endplate (SEP) in flexion (FL), extension (EX), lateral bending (LB), and axial rotation (AR) were compared among models.

**Results:** Compared with intact model and No-SPF, the ROM of global lumbopelvis, LSJ, and sacroiliac joint (SIJ) was decreased in SS-SR, MS-SR, SS-MR, and MS-MR in all directions. Compared with SS-SR, the ROM of global lumbopelvis and LSJ of MS-SR, SS-MR, and MS-MR further decreased, while the ROM of SIJ was only decreased in MS-SR and MS-MR. The stress on instrumentation, cages, S1-SEP, and sacrum decreased in SS-SR, compared with no-SPF. Compared with SS-SR, the stress in EX and AR further decreased in SS-MR and MS-SR. The most significantly decreased ROM and stress were observed in MS-MR.

**Conclusion:** Both multiple pelvic screws and multirod construct could increase the mechanical stability of LSJ and reduce stress on instrumentation, cages, S1-SEP, and sacrum. The MS-MR construct was the most adequate to reduce the risk of lumbosacral pseudarthrosis, implant failure, and sacrum fracture. This study may provide surgeons with important evidence for the application of MS-MR construct in the clinical settings.

## Introduction

Adult spinal deformity (ASD) is a heterogeneous spectrum of abnormalities causing spinal malalignment in sagittal and coronal plane (Kim et al., 2020). With prolonged life expectancy, the prevalence of ASD is up to 68% in the elderly population (Ames et al., 2016). Patients with ASD commonly complain of low back pain, radiculopathy, disability, and poor health-related quality of life (HRQoL) (Pellisé et al., 2015; Yang et al., 2023). As a reliable and lasting solution, surgical treatment for ASD has gained popularity in the last decade (Safaee et al., 2020). The primary goals of surgery are to improve HRQoL through restoration of spinal alignment and resolution of neurological deficit.

Posterior long spinal fusion was the most common surgical procedure for ASD. However, if the construct was extended to the sacrum, a high incidence of mechanical complications including pseudoarthrosis (19.0%–83.0%) and implant failure (23.7%–56.0%) has been reported due to the sacral cancellous nature, complex anatomy, and substantial shear forces at the lumbosacral junction (LSJ) (Kim et al., 2006a; Kim et al., 2006b; Kim et al., 2010; Finger et al., 2014; Guler et al., 2015; Hallager et al., 2017; Eastlack et al., 2022). Strategies for addressing this concern predominantly included anterior column support and sacropelvic fixation (SPF) to enhance the fusion rate at the LSJ and increase construct stiffness. SPF traditionally involves iliac screw or S2-alar-iliac (S2AI) screw. S2AI fixation has increased in prevalence in recent years owing to various advantages over iliac screw placements (Jain et al., 2016; Hasan et al., 2020). S2AI screw could get a stronger anchor through additional purchase in the sacrum and sacroiliac joint (SIJ). Also, S2AI was in-line with S1 screws; therefore, the need for medial-to-lateral connectors could be avoided.

Although the application of SPF, the incidence of implant failure is still unsatisfactory, with reported rates ranging from 12.0% to 46.9% (Park et al., 2021b; Gao et al., 2021). In recent cohort studies, advanced SPF technique by multiple pelvic screws or multirod construct has been recommended following long spinal fusion to stabilize the LSJ further, protect the primary rod and screws, and reduce the persistent motion of SIJ (Uotani et al., 2021; Lee et al., 2022a; Lee et al., 2022b). However, there was only one small-size cohort study reporting the application of combining the multiple pelvic screws and multirod construct, without any control groups (Shen et al., 2018). Whether this kind of construct could further decrease the risk of mechanical complications remains unknown. Understanding the biomechanical advantages of this construct could provide surgeons with some valuable guidance to solve the arising problems of mechanical complications in long spinal fusion surgery.

This was the first study to compare the biomechanical performance of combining the multiple pelvic screws and multirod construct to other advanced SPF constructs for the augmentation of LSJ in long spinal fusion surgery through finite element (FE) analysis.

## Materials and methods

### Construction of the intact FE model

A healthy 30-year male volunteer (175 cm tall and 68 kg) was recruited. History of low back pain, leg pain, spinal degeneration, deformity, infection, trauma, tumours, and abnormality of bone mass was ruled out. The study protocol was approved by the Research Ethics Committee of Beijing Chao-Yang Hospital (2022-11-02-4), and informed consent was obtained from the participant. A 128-slice spiral computed tomography (CT) scan (SOMATOM Definition AS+, Siemens, Germany) from L1 to pelvic with a thickness of 0.625 mm was performed for the participant. The tomographic images were imported into Mimics Research 21.0 (Materialise, Belgium) for three-dimensional (3D) reconstruction in Digital Imaging and Communications in Medicine format (DICOM). Through region growing, threshold segmentation, and manual mask editing, a basic 3D lumbopelvic contour model was generated and stored in STL format. Subsequently, the above data were imported into Geomagic Studio 12 (Geomagic, United States) to construct the bony contour of lumbopelvic model by smoothing, denoising, and reverse-engineering, and the geometric model was saves as STP format. Next, Hypermesh 17.0 (Altair Engineering, United States) was used for pre-processing procedures of FE analysis, including meshing, material properties assignment, definition of interaction, and application of loading and boundary conditions.

**FIGURE 1:**
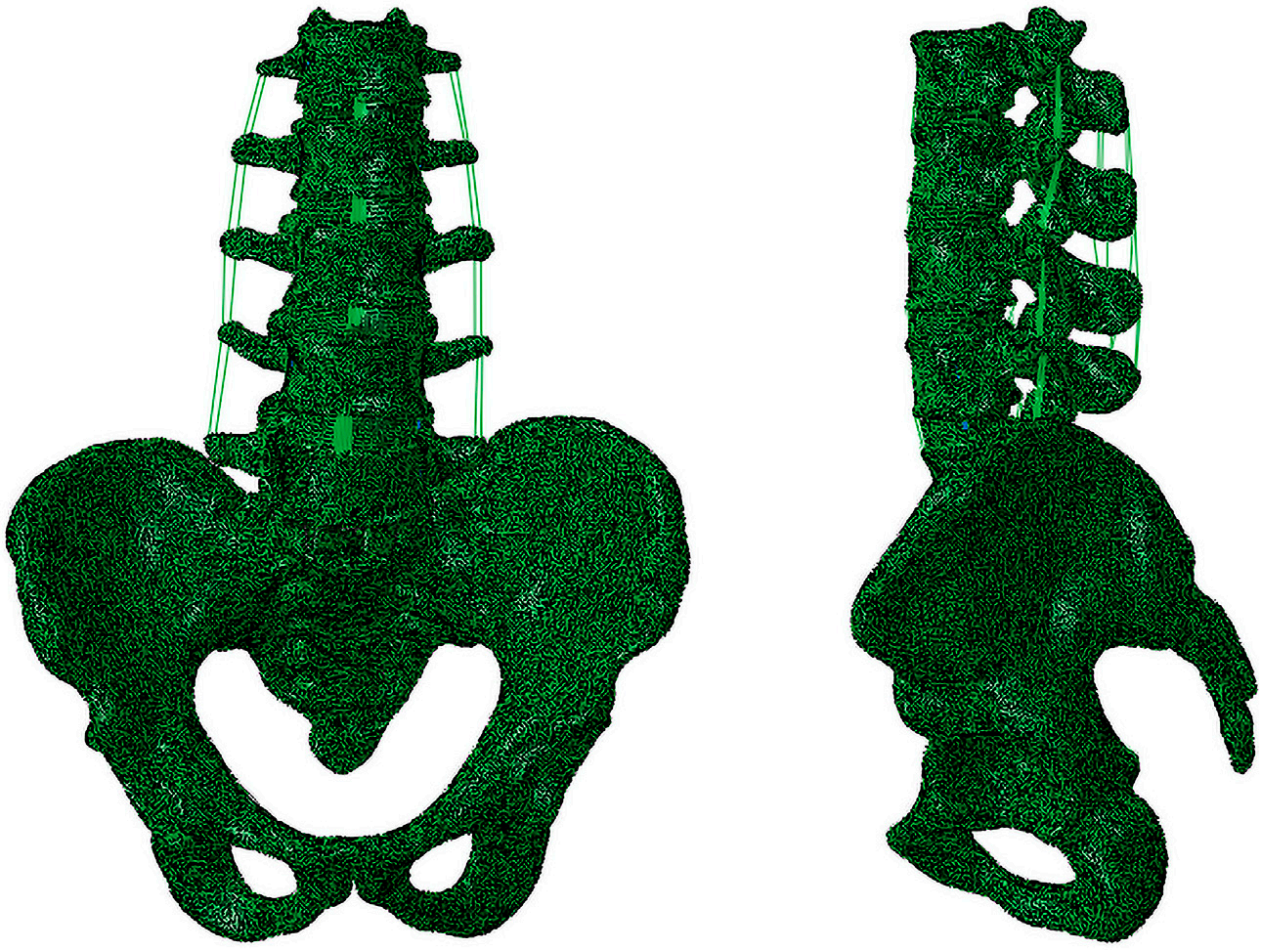
The intact lumbopelvic finite element model.

The lumbopelvic geometric model was composed of the vertebral body, intervertebral disc, and posterior elements. The vertebral body included cortical bone, cancellous bone, and cartilaginous endplates. The intervertebral disc was consisted of nucleus pulposus and annulus fibrosus, a ground matrix reinforced by fibres. The thickness of the cortical bone and endplate was set as 1.0 mm and 0.5 mm, respectively. The nucleus pulposus accounted for around 50% of the intervertebral disc volume, and the thickness of the articular cartilage was assumed to be 0.2 mm. A frictionless surface contact between facet joints was assigned, and the SIJ interaction was modelled as surface-to-surface contact with a frictional coefficient of 0.4 (Kiapour et al., 2020). Ligaments included anterior longitudinal ligament, posterior longitudinal ligament, ligamentum flavum, capsular ligament, intertransverse ligament, interspinous ligament, supraspinous ligament, anterior sacroiliac ligament, posterior sacroiliac ligament, interosseous sacroiliac ligament, sacrospinous ligament, sacrotuberous ligament, superior pubic ligament, arcuate pubic ligament, inguinal ligament, and the iliolumbar ligament were generated using hyper-elastic, tension-only, two-node Truss elements (T3D2). The insertion locations of ligaments were referenced from the anatomical attachment points. The intact FE model included 173,636 nodes and 738,423 elements (Figure 1). The properties of all components in the lumbopelvic model were listed in Table 1 and Table 2, according to the literature (Hakim and King, 1979; Shirazi-Adl et al., 1984; Dalstra and Huiskes, 1995; Zheng et al., 1997; Kawahara et al., 2003; Rohlmann et al., 2006; Phillips et al., 2007; Schmidt et al., 2007; Shi et al., 2014; Sohn et al., 2018).

**TABLE 1:** Material properties of the lumbopelvic finite element model.

Components	Young’s modulus (MPa)	Poisson’s ratio	Element type	Reference
Lumbar vertebra cortical bone	12000	0.30	C3D8R	Kawahara et al. (2003)
Lumbar vertebra cancellous bone	100	0.30	C3D4	
Sacrum cortical bone	6140	0.30	C3D6	Hakim and King (1979)
Sacrum cancellous bone	1400	0.30	C3D4	
Ilium cortical bone	17000	0.30	C3D6	Dalstra and Huiskes (1995)
Ilium cancellous bone	132	0.20	C3D4	
Sacrum cartilage	54	0.40	C3D8H	Sohn et al. (2018)
Ilium cartilage	54	0.40	C3D8H	
Pubic symphysis	5	0.45	C3D10	Shi et al. (2014)
Endplate	100	0.30	C3D4	Shirazi-Adl et al. (1984)
Annulus fiber	450	0.30	T3D2	
Annulus matrix	C_10_ = 0.18, C_01_ = 0.045	0.30	C3D8H	Schmidt et al. (2007)
Nucleus pulposus	C_10_ = 0.12, C_01_ = 0.03	0.50	C3D8H	

**TABLE 2:** Material properties of the lumbar and pelvic ligaments.

Ligament	Stiffness coefficient (N/mm)	Reference
Anterior longitudinal ligament	1864	Rohlmann et al. (2006)
Posterior longitudinal ligament	236	
Ligamentum flavum	58	
Capsular ligament	384	
Intertransverse ligament	11	
Interspinous ligament	15	
Supraspinous ligament	34	
Anterior sacroiliac ligament	700	Zheng et al. (1997)
posterior sacroiliac ligament	400	
Interosseous sacroiliac ligament	2800	
Sacrospinous ligament	1400	
Sacrotuberous ligament	1500	
Superior pubic ligament	500	
Arcuate pubic ligament	500	
Inguinal ligament	250	Phillips et al. (2007)
Iliolumbar ligament	1000	

### Generation of the instrumented model

**FIGURE 2:**
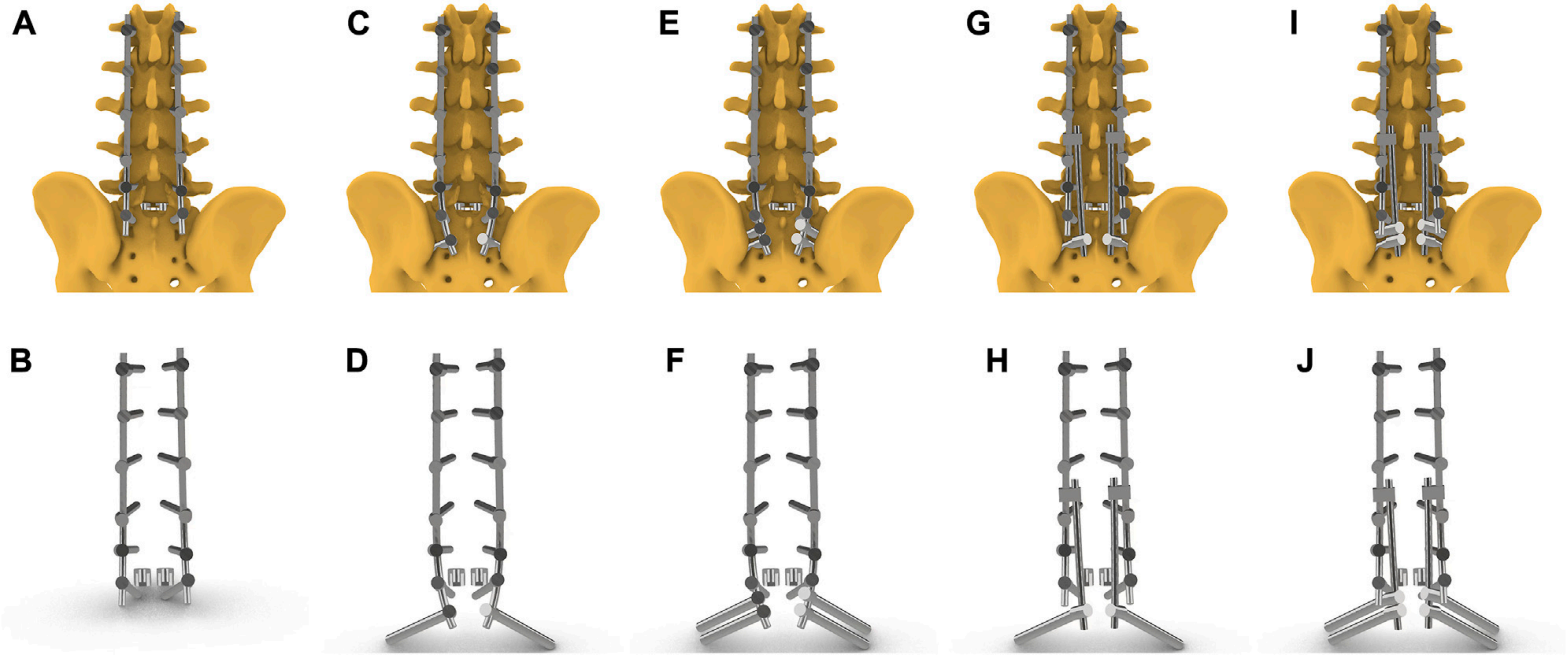
Five instrumented models including instrumentations and cages. **(A, B)** No-SPF: SPF was not performed and only bilateral pedicle screws were inserted at S1; **(C, D)** SS-SR: The primary rod was anchored to single S2AI screw, and there was no any accessory rods. **(E, F)** MS-SR: The primary rod was anchored to dual S2AI screws, and there was no any accessory rods; **(G, H)** SS-MR: The primary rod was anchored to S1-PS. Medial accessory rod was used, with distal end anchored to single S2AI screw and proximal end connected to the ipsilateral primary rod by rod-rod connector; **(I, J)** The primary rod was anchored to S1-PS. Medial accessory rod was used, with distal end anchored to dual S2AI screws and proximal end connected to the ipsilateral primary rod by rod-rod connector.

The instrumented model was posterior bilateral pedicle screw fixation from L1 to S1 with posterior lumbar interbody fusion (PLIF) and SPF. There was no any facetectomy, laminectomy, or discectomy from L1 to L4. Regarding to PLIF, resections of the spinous processes, laminectomy, and inferior facetectomy were performed at L5. The intervertebral disc and endplates of L5/S1 were removed, and two cube-shaped fusion cages were implanted. The instrumentation for SPF was different among the instrumented models (Figure 2).

(1) No-SPF: SPF was not performed and only bilateral pedicle screws were inserted at S1.
(2) Bilateral single S2AI screw and single rod (SS-SR): The primary rod was anchored to single S2AI screw, and there was no any accessory rods.
(3) Bilateral multiple S2AI screws and single rod (MS-SR): The primary rod was anchored to dual S2AI screws, and there was no any accessory rods.
(4) Bilateral single S2AI screw and multiple rods (SS-MR): The primary rod was anchored to S1-PS. Medial accessory rod was used, with distal end anchored to single S2AI screw and proximal end connected to the ipsilateral primary rod by rod-rod connector.
(5) Bilateral multiple S2AI screws and multiple rods (MS-MR): The primary rod was anchored to S1-PS. Medial accessory rod was used, with distal end anchored to dual S2AI screws and proximal end connected to the ipsilateral primary rod by rod-rod connector.

SolidWorks (Dassault Systems, United States) was used to design and assemble the screws, rods, cages, and connecters in instrumented models. The rods were simulated by fitting lines passing though centres of screw caps. C3D8R was applied to mesh these implants. Ti6Al4V and PEEK were assigned to the materials of the posterior instrumentation and cages, respectively. The contact surface of screw-rod, screw-vertebral body, and cage-endplate were set as tie constraints.

### Validation of the intact FE model

The ROM of each lumbar segment and SIJ in this intact FE model was compared to the data in several *in vitro* studies under equivalent loading conditions (Panjabi et al., 1994; Lindsey et al., 2014; Cook et al., 2015; Cross et al., 2018; Ntilikina et al., 2020; Sayed et al., 2021). For the validation of ROM of each lumbar segment, the S1 was constrained, and pure moments of 7.5 Nm in flexion (FL), extension (EX), lateral bending (LB), and axial rotation (AR) were applied to the superior endplate (SEP) of L1. For the validation of ROM of SIJ, the right ilium was fixed, and pure moments of 7.5 Nm in six directions were applied to L4-SEP.

The intradiscal pressure (IDP) of each lumbar segment was compared to the data in a cadaveric test by Hsiao et al. (Hsiao et al., 2022). Pure moments of 7.5 Nm with and without an axial load of 500 N in FL, EX, and LB were applied to the superior endplate of L1.

### Validation of the instrumented FE models

As MS-SR, SS-MR, and MS-MR were relatively novel instrumented models, no cadaveric study using these three models was reported. There were two cadaveric studies using the instrumented models of No-SPF and SS-SR, and both were performed by Pereira et al. (de Andrada Pereira et al., 2021; de Andrada Pereira et al., 2022). After carefully reviewing the cadaveric specimen information in these studies, we confirmed that the cadaveric specimen were not reused. Only anterior lumbar interbody fusion was performed at L5/S1 in the cadaveric instrumented models by Pereira et al., without any laminectomy or facetectomy, which may impact the stability and stress distribution of spine. To make our validation more reliable, we restored the lamina and facet joints but the interbody fusion cages were preserved before pure moments of 7.5 Nm were applied.

The ROM of L2-S1 and LSJ in the No-SPF and SS-SR models was compared to the data in the studies by Pereira et al. (de Andrada Pereira et al., 2021; de Andrada Pereira et al., 2022). The rod strains on lumbosacral rod (between L5-PS and S1-PS) and S1-S2 rod were also validated. Consistent with the protocol by Pereira et al., the rod trains were measured on the posterior surface, at the middle level, on the right side.

### Loading and boundary condition

The loads and boundary conditions were set in Abaqus 6.10 (Dassault Systems, France) for FE analysis. In all the FE models, the iliac was fully constrained in all degrees of freedom. A load of 500 N and a pure moment of 7.5 Nm was applied to the nodes coupled with L1-SEP to simulate flexion, extension, lateral bending and axial rotation under the physiological compressive load

### Data analysis

The global ROM, the ROM of LSJ, the maximum von-Mises stress (VMS) on instrumentation, the S1-PS, the lumbosacral rods, the cages, the sacrum, and S1-SEP in FL, EX, LB, and AR were compared among the intact model and instrumented models.

## Results

### Validation of the intact and instrumented FE models

**FIGURE 3:**
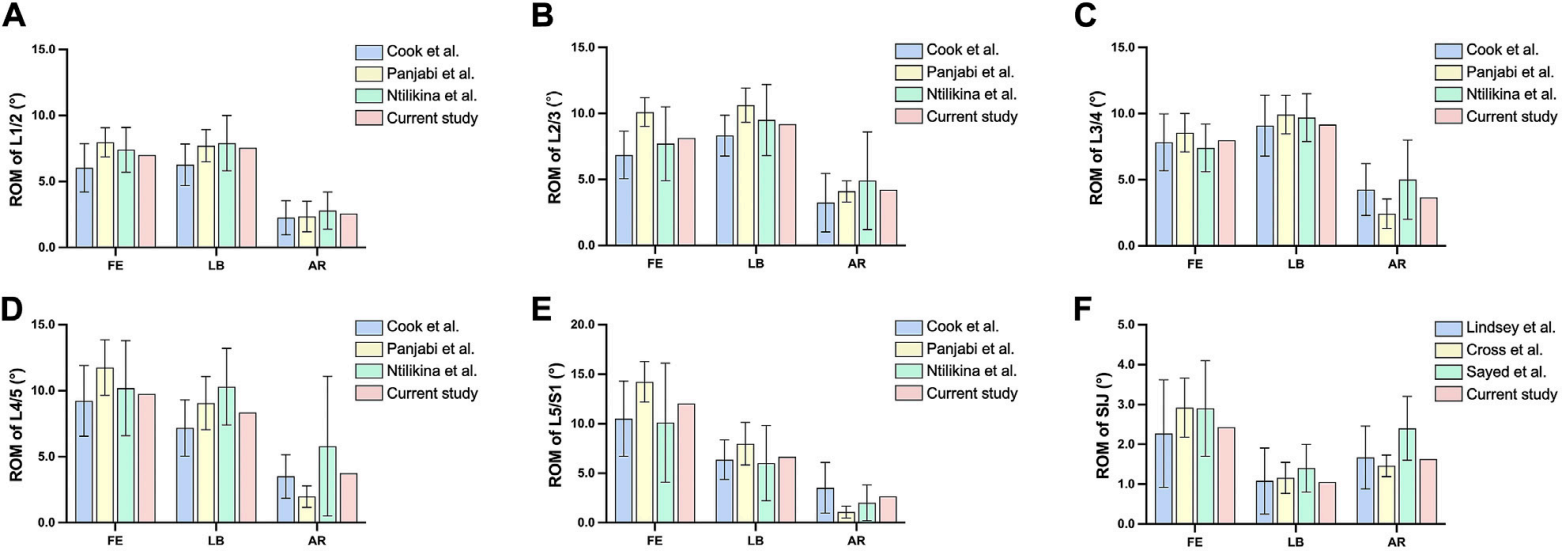
Comparison of the range of motion between the intact model and *in vitro* studies. **(A)** range of motion of L1/2; **(B)** range of motion of L2/3; **(C)** range of motion of L3/4; **(D)** range of motion of L4/5; **(E)** range of motion of L5/S1; **(F)** range of motion of sacroiliac joint.

The ROM of each lumbar segment and SIJ in the current intact FE model was consistent with the data from the literature (Figure 3). The IDP of each lumbar segment in the intact FE model was also consistent with the data from the study by Hsiao et al. (Supplementary Figure S1). (Hsiao et al., 2022). The ROM of L2-S1 and LSJ in the No-SPF and SS-SR instrumented models was consistent with the data from the studies by Pereira et al. (Supplementary Figure S2). (de Andrada Pereira et al., 2021; de Andrada Pereira et al., 2022). The rod strains on lumbosacral rod and S1-S2 rod were also well validated (Supplementary Figure S3). The validations suggested that the intact and instrumented lumbopelvic models in the present study were effective and reliable, which could be used for further analysis.

### Global ROM

**FIGURE 4:**
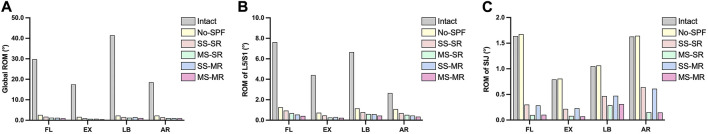
Comparison of the range of motion among intact and instrumented models. **(A)** the global range of motion; **(B)** the range of motion of lumbosacral junction; **(C)** the range of motion of sacroiliac joint.

The global ROM of intact model, No-SPF, SS-SR, MS-SR, SS-MR, and MS-MR in all directions was demonstrated in Figure 4A. Compared with intact model, the global ROM of No-SPF decreased in all directions; compared with No-SPF, the ROM of SS-SR decreased by 32.97%, 39.25%, 31.43%, and 38.21% in FL, EX, LB, and AR, respectively. Compared with SS-SR, the ROM further decreased in MS-SR, SS-MR, and MS-MR. The most significant decreased ROM was observed in MS-MR, ranging from 27.30% in LB to 54.21% in EX, compared with SS-SR. In FL, EX, and AR, the ROM was similar between MS-SR and SS-MR; however, in LB, the ROM was similar between SS-SR and SS-MR, as well as between MS-SR and MS-MR.

### ROM of lumbosacral junction

The ROM of LSJ of intact model, No-SPF, SS-SR, MS-SR, SS-MR, and MS-MR in all directions was demonstrated in Figure 4B. Compared with intact model, the ROM of No-SPF decreased in all directions; compared with No-SPF, the ROM of SS-SR decreased by 25.81%, 37.62%, 32.81%, and 36.18% in FL, EX, LB, and AR, respectively. Compared with SS-SR, the ROM further decreased in MS-SR, SS-MR, and MS-MR. The most significant decreased ROM was observed in MS-MR, ranging from 43.25% in LB to 55.29% in FL, compared with SS-SR. The ROM of SS-MR decreased by 20.71% in FL and 13.91% in AR but increased by 17.00% in EX, compared with MS-SR. In LB, the ROM was similar between MS-SR and SS-MR.

### ROM of sacroiliac joint

The ROM of SIJ of intact model, No-SPF, SS-SR, MS-SR, SS-MR, and MS-MR in all directions was demonstrated in Figure 4C. Compared with intact model, the ROM of No-SPF slightly increased in all directions. Compared with No-SPF, the ROM of SS-SR decreased by 82.03%, 73.36%, 55.83%, and 61.07% in FL, EX, LB, and AR, respectively. Compared with SS-SR, the ROM of MS-SR further decreased by 68.11%, 62.33%, 38.30%, and 76.09% in FL, EX, LB, and AR, respectively. The ROM was similar between SS-SR and SS-MR, as well as between MS-SR and MS-MR.

### Maximum von-Mises stress on instrumentation

**FIGURE 5:**
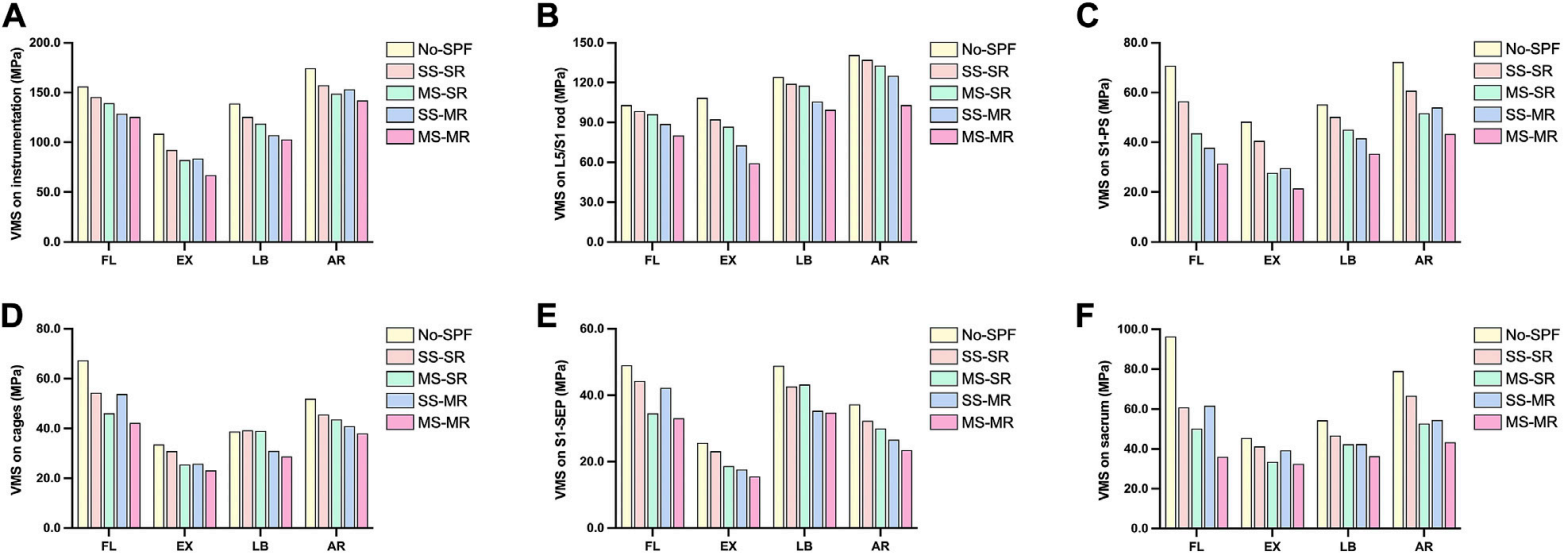
Comparison of the maximum von-Mises stress among instrumented models. **(A)** instrumentation; **(B)** lumbosacral rods; **(C)** S1-pedicle screws; **(D)** cages; **(E)** S1-superior endplate; **(F)** sacrum.

**FIGURE 6:**
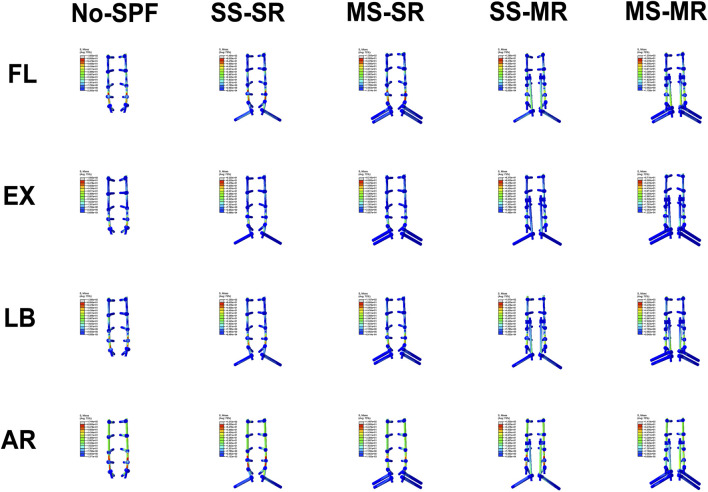
Von-Mises stress on instrumentation of different instrumented models in flexion, extension, lateral bending, and axial rotation.

The maximum VMS on instrumentation in No-SPF, SS-SR, MS-SR, SS-MR, and MS-MR in all directions was demonstrated in Figure 5A and Figure 6. Compared with No-SPF, the maximum VMS on SS-SR decreased by 6.79%, 14.93%, 9.71%, and 9.79% in FL, EX, LB, and AR, respectively. Compared with SS-SR, the maximum VMS further decreased in MS-SR, SS-MR, and MS-MR in all directions. The most significantly decreased maximum VMS was observed in MS-MR, ranging from 9.82% in AR to 27.30% in EX, compared with SS-SR. In FL and LB, the maximum VMS gradually decreased from MS-SR to MS-MR; however, in EX and AR, the VMS on SS-MR was slightly greater than MS-SR.

#### Maximum von-Mises stress on lumbosacral rods

The maximum VMS on lumbosacral rods in No-SPF, SS-SR, MS-SR, SS-MR, and MS-MR in all directions was demonstrated in Figure 5B. Compared with No-SPF, the maximum VMS on SS-SR significantly decreased in EX. The maximum VMS was similar between SS-SR and MS-SR in all directions; however, the maximum VMS on both SS-MR and MS-MR were significantly lower than MS-SR. The most significantly decreased maximum VMS was observed in MS-MR, ranging from 16.54% in LB to 35.85% in EX, compared with SS-SR. Compared with SS-MR, the maximum VMS on MS-MR further decreased by 9.64%, 18.44%, 5.90%, and 17.67% in FL, EX, LB, and AR, respectively.

#### Maximum von-Mises stress on S1-PS

The maximum VMS on S1-PS in No-SPF, SS-SR, MS-SR, SS-MR, and MS-MR in all directions was demonstrated in Figure 5C. Compared with No-SPF, the maximum VMS on SS-SR decreased by 20.09%, 16.06%, 8.98%, and 15.97% in FL, EX, LB, and AR, respectively. Compared with SS-SR, the maximum VMS further decreased in MS-SR, SS-MR, and MS-MR. The most significantly decreased maximum VMS was observed in MS-MR, ranging from 28.71% in AR to 47.03% in EX, compared with SS-SR. In FL and LB, the maximum VMS gradually decreased from MS-SR to MS-MR; however, in EX and AR, the VMS on SS-MR was slightly greater than MS-SR. This trend was similar to that of global instrumentation.

#### Maximum von-Mises stress on cages

**FIGURE 7:**
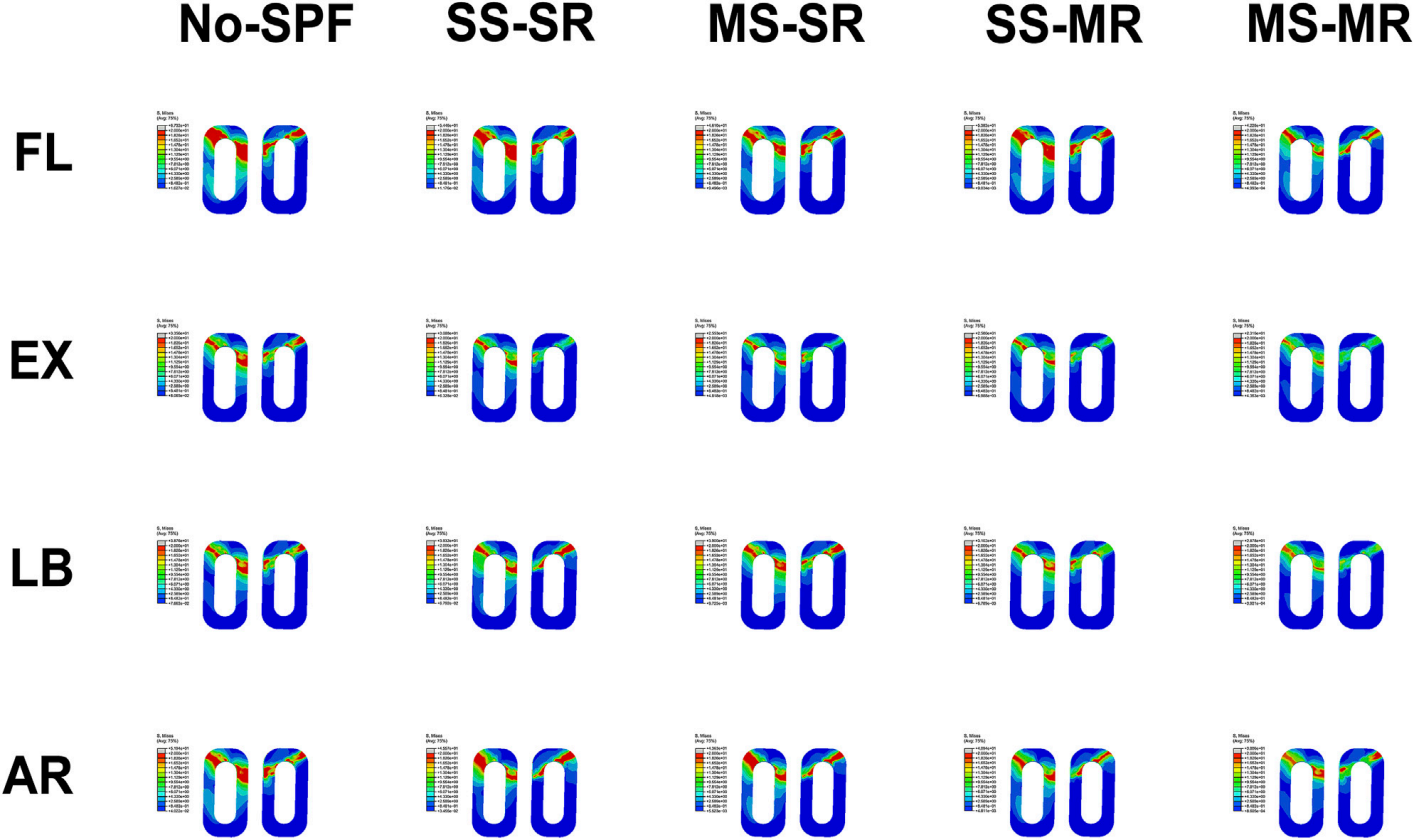
Von-Mises stress on cages of different instrumented models in flexion, extension, lateral bending, and axial rotation.

The maximum VMS on cages in No-SPF, SS-SR, MS-SR, SS-MR, and MS-MR in all directions was demonstrated in Figure 5D and Figure 7. Compared with No-SPF, the maximum VMS on SS-SR decreased in FL, EX, and AR. Compared with SS-SR, in FL, the maximum VMS significantly decreased in MS-SR and MS-MR but comparative in SS-MR; in EX, the maximum VMS significantly decreased in MS-SR, SS-MR, and MS-MR; in LB, the maximum VMS significantly decreased in SS-MR and MS-MR but comparative in MS-SR; in AR, the maximum VMS gradually decreased from MS-SR to MS-MR. The most significantly decreased maximum VMS was observed in MS-MR, ranging from 16.48% in AR to 26.81% in LB, compared with SS-SR. The maximum VMS in EX was similar between MS-SR and SS-MR.

#### Maximum von Mises stress on S1-SEP

**FIGURE 8:**
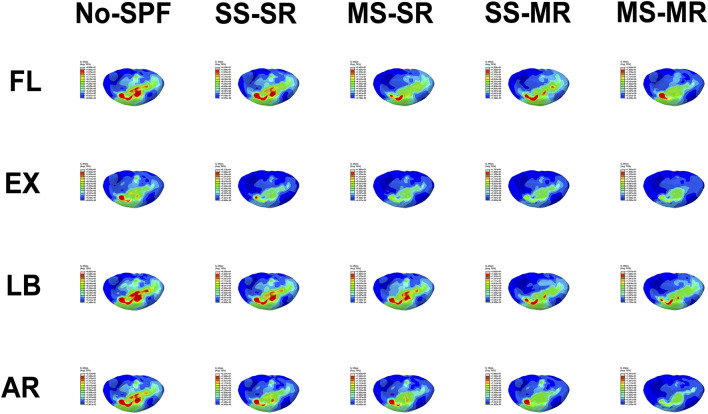
Von-Mises stress on S1-superior endplate of different instrumented models in flexion, extension, lateral bending, and axial rotation.

The maximum VMS on S1-SEP in No-SPF, SS-SR, MS-SR, SS-MR, and MS-MR in all directions was demonstrated in Figure 5E and Figure 8. Compared with No-SPF, the maximum VMS on SS-SR decreased by 9.62%, 9.90%, 12.67%, and 13.18% in FL, EX, LB, and AR, respectively. Compared with SS-SR, in FL, the maximum VMS significantly decreased in MS-SR and MS-MR but comparative in SS-MR; in EX, the maximum VMS significantly decreased in MS-SR, SS-MR, and MS-MR; in LB, the maximum VMS significantly decreased in SS-MR and MS-MR but comparative in MS-SR; in AR, the maximum VMS gradually decreased from MS-SR to MS-MR. This trend was consistent with that of cages. The most significantly decreased maximum VMS was observed in MS-MR, ranging from 18.50% in LB to 32.83% in EX, compared with SS-SR.

### Maximum von Mises stress on sacrum

**FIGURE 9:**
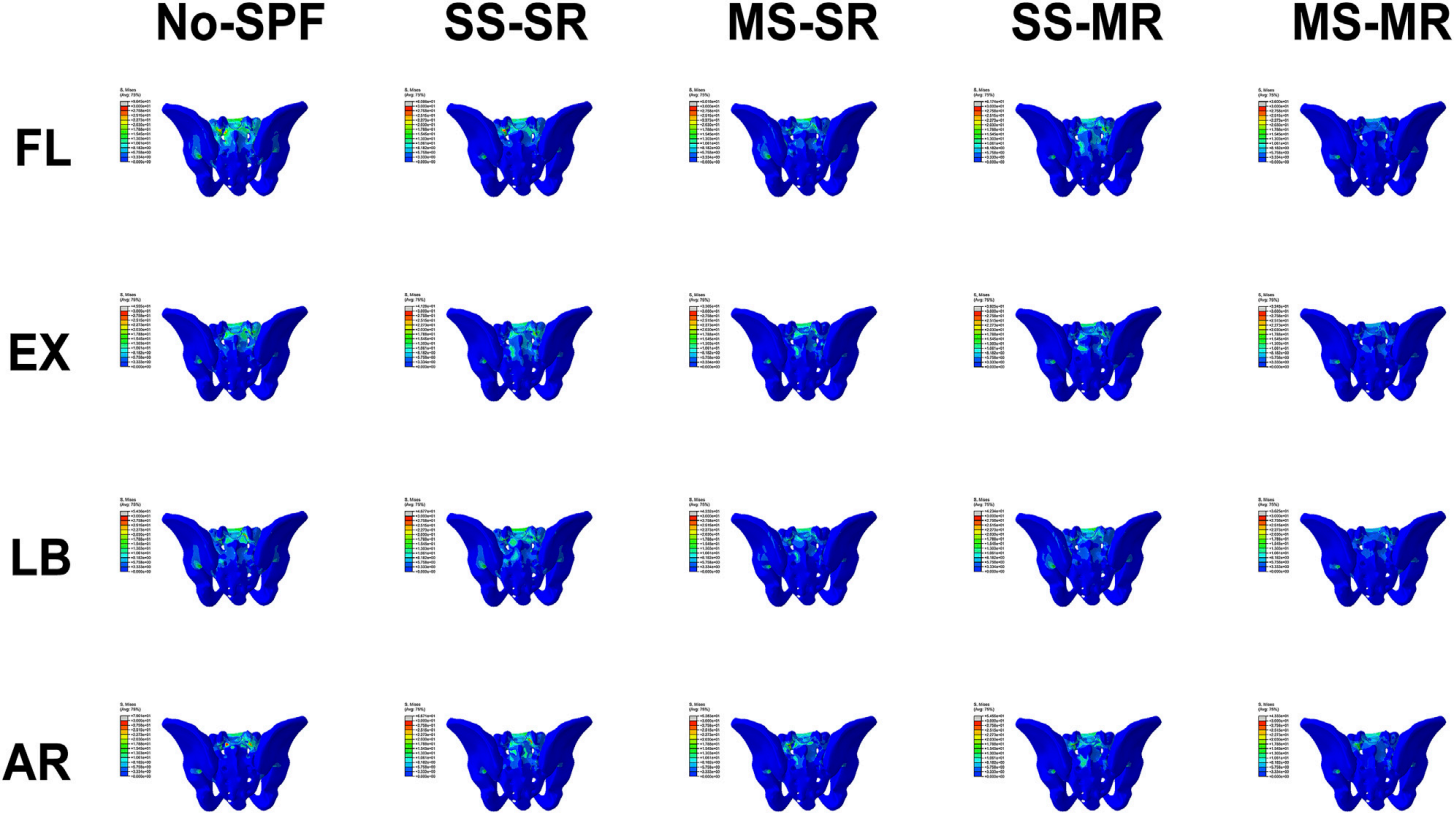
Von-Mises stress on sacrum of different instrumented models in flexion, extension, lateral bending, and axial rotation.

The maximum VMS on the sacrum in No-SPF, SS-SR, MS-SR, SS-MR, and MS-MR in all directions was demonstrated in Figure 5F and Figure 9. Compared with No-SPF, the maximum VMS on SS-SR decreased by 36.90%, 9.37%, 13.96%, and 15.57% in FL, EX, LB, and AR, respectively. Compared with SS-SR, in EX, LB, and AR, the maximum VMS further decreased in MS-SR, SS-MR, and MS-MR; however, in FL, the maximum VMS was comparative in SS-MR. The most significantly decreased maximum VMS was observed in MS-MR, ranging from 21.32% in EX to 40.85% in FL, compared with SS-SR. Compared with SS-MR, the maximum VMS on MS-MR further decreased by 41.69%, 17.25%, 14.38%, and 20.58% in FL, EX, LB, and AR, respectively.

## Discussion

The substantial biomechanical shear forces of LSJ, poor bone quality of sacrum, and complex sacral anatomy make long spinal fusion challenging to achieve solid lumbosacral fusion, which impacts patients’ HRQoL and usually needs revision surgery (Kim et al., 2010). Also, the large lever arms and cantilever forces result in high stress on the base of the construct, increasing the risk of implant failure. Therefore, SPF has been proposed following long spinal fusion ending at S1 (Guler et al., 2015; El Dafrawy et al., 2019). Although SPF indeed reinforces the construct stiffness, 12.0%–46.9% of implant failure rate has been reported by previous studies (Park et al., 2021b; Gao et al., 2021). To reduce the incidence of mechanical complications, multiple pelvic screws or multirod construct have been applied as an advanced SPF technique (Uotani et al., 2021; Lee et al., 2022a; Lee et al., 2022b; Tang et al., 2022). The current study revealed that both multiple pelvic screws and multirod construct could increase the mechanical stability of LSJ, reduce strain on the lumbosacral rod, and protect the S1-PS and sacrum. The combination of these two constructs could further improve these effects. This study may play a role of pre-clinical evaluation of MS-MR construct and could provide surgeons with more information about its advantages over the other advanced SPF constructs.

The LSJ is the most mobile and anteriorly inclined segment in the lumbar spine (Park et al., 2021a). These biomechanical characteristics induce a risk of pseudarthrosis in this region, which could predispose the implant to mechanical failure (Park et al., 2021b). Several studies reported a high incidence of lumbosacral pseudarthrosis with S1-PS alone (19.0%–83.0%) or additional SPF (10.5%–33.3%) (Kim et al., 2006a; Kim et al., 2006b; Finger et al., 2014; Guevara-Villazón et al., 2020; Eastlack et al., 2022). A meta-analysis by Han et al. reported that there was no significant difference in the pseudarthrosis rate between patients with or without SPF (Han et al., 2021). Therefore, advanced SPF technique by multiple pelvic screws or multirod construct was tried to solve this problem. Cadaveric studies have shown that four-rod constructs could significantly reduce the flexibility and motion of LSJ in FL, EX, and AR (Kelly et al., 2008; Wang et al., 2013; Godzik et al., 2019; Ntilikina et al., 2020). Consistent with previous studies, the current study additionally indicated that both the multiple pelvic screws and multirod construct could decrease the ROM of LSJ in FL, EX, LB, and AR compared with the SS-SR model. The ROM decreased most in the MS-MR model. In this construct, there were four rods crossing the LSJ, and the caudal anchors of the four rods were independent, which may contribute to its superior biomechanical characteristics. Therefore, we considered that additional pelvic screws and accessory rods had the potential to stabilize the LSJ further and achieve solid fusion. Priority should be given to the MS-MR construct for patients with high risks of pseudarthrosis.

Anterior column support using fusion cages could enhance the fusion rate and prevent implant failure at LSJ (Jung et al., 2019; Lee et al., 2020). However, osteoporosis is a risk factor for cage subsidence, with a high prevalence of 32.8% in ASD patients undergoing long spinal fusion (Gupta et al., 2021; Yang et al., 2021). Therefore, an appropriate biomechanical environment is paramount to a solid interbody fusion for ASD patients. The current study detected similar trends in the VMS variation on cage and S1-SEP. Compared with No-SPF and SS-SR, the VMS decreased the most in the MS-MR model. The VMS on cages in all directions decreased, ranging from 25.75% to 37.23% compared with No-SPF and 16.48%–26.81% compared with SS-SR; the VMS on S1-SEP in all directions decreased, ranging from 28.82% to 39.48% compared with No-SPF and 18.50%–32.83% compared with SS-SR. This finding indicated that the MS-MR construct could facilitate lumbosacral fusion by reducing the risk of cage subsidence. For patients with risk factors of cages subsidence, such as osteoporosis and age >60 years, the MS-MR could be considered.

The high incidence of lumbosacral and pelvic implant failure is a major concern for long spinal fusion. Substantial rates ranging from 23.7% to 56.0% have been reported by previous cohort studies (Guler et al., 2015; Hallager et al., 2017; Eastlack et al., 2022). Among the implant failure, rod fracture (RF) was the most common complication, and 28.0%–81.3% of RF occurred at lumbosacral rods (Devlin et al., 1991; Lertudomphonwanit et al., 2018; Rabinovich et al., 2021). The culprits of RF were pseudarthrosis and the increasing fatigue cracks under external stress (Yamanaka et al., 2015; Sardi et al., 2022). We reviewed and synthesised the data of all published clinical studies comparing the mechanical complication rate between two-rod construct and multirod construct, and a significantly lower incidence of pseudarthrosis (14.2% vs. 35.0%) and RF (15.8% vs. 32.9%) was detected in patients undergoing multirod construct (Hyun et al., 2014; Han et al., 2017; Merrill et al., 2017; Guevara-Villazón et al., 2020; Yamato et al., 2020; Bourghli et al., 2021; Dinizo et al., 2021; Lamas et al., 2021; Rabinovich et al., 2021; Lyu et al., 2022). There are two options to anchor the distal end of accessory rods, including domino connectors and multiple pelvic screws. The way of stress dispersal was different between the two options. Domino connector is the most popular option, in which the accessory rods were anchored to the primary rods instead of the vertebra, just as satellite rods. The stress of primary rods was transferred to the accessory rods, but it would be finally transferred back at the region distal to the accessory rods, resulting in RF or screw breakage at LSJ (Palumbo et al., 2015; Shen et al., 2018). However, by multiple pelvic screws, the accessory rods were directly anchored to the pelvis. The four rods crossing the LSJ are more mechanically independent, similar to two separate spinal constructs, providing the majority of cantilever force (Ramey et al., 2021). Cadaveric studies by Ntilikina et al. (2020) and Godzik et al. (2019) reported that accessory rods connected by domino connector could significantly decrease the strain of primary rods in FL, EX, LB, and the strain of S1-PS in AR. Nevertheless, the protective effect of accessory rods connected by multiple pelvic screws is still unknown. VMS was used in the current study. VMS is an equivalent stress value based on distortion energy to decide if a material will fail (yield) under a given loading condition. For spine and spinal instrumentation, a higher VMS on bone or internal fixation suggests a greater amount of deformation on it, which makes it more prone to instrumentation failure or fracture. We found that the VMS on instrumentation was decreased with the additional use of either S2AI screws or accessory rods. The VMS mainly concentrated on the L5-S1 rod, and the MS-MR model decreased the VMS in all directions, especially in EX, compared with No-SPF (45.40%) and SS-SR (35.85%). Also, both multiple pelvic screws and multirod constructs could protect the S1-PS and sacrum, and this effect provided by the MS-MR model was the most significant. This finding indicated that MS-MR could enhance the construct stiffness, reduce the risk of RF and screw breakage at LSJ, and reduce the risk of sacrum fracture. Additionally, the less stress values on MS-MR construct may indicate its superior durability, which could provide patients with long-term benefits.

The persistent motion of SIJ may result in further stress on the SPF and induce delayed failures (Eastlack et al., 2022). Tang et al., 2022 reported that the dual S2AI screws could mitigate postoperative SIJ pain and play an anti-rotation role. Consistent with their study, the current study revealed that the additional S2AI screw could further decrease the ROM of SIJ, especially in AR. This advantage may be associated with reduced VMS on instrumentation.

In addition to pseudarthrosis, postoperative residual sagittal and/or coronal malalignment are risk factors for implant failure following long spinal fusion for ASD (Lee et al., 2022b; Martin et al., 2022). For complex cases with preoperative several sagittal and coronal malalignment, three-column osteotomy (3-CO) and sequential correction techniques are usually needed (Lau et al., 2021; Shi et al., 2021). Therefore, a secure foundation and stiff construct that could accommodate powerful deformity correction and maintenance are essential. The dual pelvic screws could provide a stronger pelvic anchorage, and the accessory rods could reinforce the spinal construct, facilitating the restoration of spinopelvic alignment. For patients who need undergoing 3-CO at lumbar vertebra and extending the instrumentation into the upper thoracic region, some modifications could be made to the MS-MR construct presented in this study: the distal end of the primary rods should be extended and anchored to S2/ilium by pelvic screws; the proximal end of the accessory rods should be anchored to the distal adjacent vertebra of the upper instrumented vertebra by PS or cortical bone trajectory screw. More importantly, all the four rods should cross both the LSJ and the osteotomy site. These modifications made the four rods completely mechanically independent, constructing two separate spinopelvic fixation constructs. According to the spine instrumentation nomenclature provided by Ramey et al. the accessory rods in this kind of construct should be defined as “secondary rods” (Ramey et al., 2021). S2AI screw and iliac screw are two commonly used pelvic screws to achieve SPF. However, various advantages of S2AI screw over iliac screw have been demonstrated. The systematic review of biomechanical studies by Hirase et al. suggested that the stress on instrumentations and surrounding iliac bone was lower in S2AI screw fixation than iliac screw (Hirase et al., 2022). The meta-analysis by Gao et al. reported that using S2AI screw could adequately maintain the deformity correction and significantly decrease the risk of mechanical complications compared with the iliac screw (Gao et al., 2021). Also, the placement of S2AI screw does not require dissection as extensive as iliac screw; therefore, the incidence of skin breakdown and would infection was lower. Accordingly, when surgeons decide to implant multiple pelvic screws, we advocated S2AI screw as routine instrumentation.

The current biomechanical research may facilitate surgeons to better understand the mechanism of mechanical complications in long spinal fusion surgery and improve the instrumentation scenario or device designs. However, several limitations should be noted. First, as constructing a real ASD patient-specific lumbopelvic model and simulating the correction procedures (e.g., multi-level decompression, laminectomy, facetectomy, or osteotomies) remain technically challenging, the FE model was developed using the CT images from a healthy volunteer as an alternative. Therefore, the abnormal spinal loading caused by ASD and the individual variation of degeneration were not considered. This methodology was consistent with the previous FE studies focusing on the performance of different instrumentations and instrumentation-related problems in ASD (Buell et al., 2019; He et al., 2021; Oe et al., 2021; Leszczynski et al., 2022; Son et al., 2022). Further studies should construct and use ASD patient-specific FE models to elucidate the clinical significance of the various instrumentations. Second, boundary conditions were assigned based on the literature. However, the actual mechanical environment was patient-specific. Therefore, a discrepancy may exist between the FE model and clinical observations. Third, some simplifications were made in the construction of FE models. The paraspinal muscles and surrounding soft tissues were not simulated, and the nucleus pulposus was assigned with a hyperplastic material, which may affect the flexibility of the spine and misrepresent the stress distribution on some spinal components. Biphasic materials composed of a solid phase embedded in a fluid media may be more appropriate and realistic for the modelling of intervertebral disc (Elmasry et al., 2017). Also, bone mineral density could influence the stress pattern in SEP and cage, but it was not considered a variable in the current study. Finally, although FE analysis has the advantage of eliminating anatomical variability and has been shown to be a reliable method to perform biomechanical comparisons among spinal instrumentations, the actual clinical effects of MS-MR construct still need to be verified by cadaveric tests as well as clinical trials with large sample size and long-term follow-up.

## Conclusion

Both multiple pelvic screws and multirod construct could increase the mechanical stability of LSJ and reduce stress on construct, cages, S1-SEP, and sacrum. The MS-MR construct was the most adequate to reduce the risk of lumbosacral pseudarthrosis, RF, screw breakage, and sacrum fracture. This study may provide surgeons with important evidence for the application of MS-MR construct in the clinical settings.

## Data Availability

The raw data supporting the conclusion of this article will be made available by the authors, without undue reservation.

## References

[B1] Ames C. P. Scheer J. K. Lafage V. Smith J. S. Bess S. Berven S. H. (2016). Adult spinal deformity: Epidemiology, health impact, evaluation, and management. Spine Deform. 4 (4), 310–322. 10.1016/j.jspd.2015.12.009 27927522

[B2] Bourghli A. Boissiere L. Kieser D. Larrieu D. Pizones J. Alanay A. (2021). Multiple-rod constructs do not reduce pseudarthrosis and rod fracture after pedicle subtraction osteotomy for adult spinal deformity correction but improve quality of life. Neurospine 18 (4), 816–823. 10.14245/ns.2142596.298 34670073 PMC8752720

[B3] Buell T. J. Bess S. Xu M. Schwab F. J. Lafage V. Ames C. P. (2019). Optimal tether configurations and preload tensioning to prevent proximal junctional kyphosis: A finite element analysis. J. Neurosurg. Spine 30, 574–584. 10.3171/2018.10.Spine18429 30738393

[B4] Cook D. J. Yeager M. S. Cheng B. C. (2015). Range of motion of the intact lumbar segment: A multivariate study of 42 lumbar spines. Int. J. Spine Surg. 9, 5. 10.14444/2005 25785241 PMC4360610

[B5] Cross W. W. 3rd Berven S. H. Slater N. Lehrman J. N. Newcomb A. Kelly B. P. (2018). *In vitro* biomechanical evaluation of a novel, minimally invasive, sacroiliac joint fixation device. Int. J. Spine Surg. 12 (5), 587–594. 10.14444/5072 30364882 PMC6198630

[B6] Dalstra M. Huiskes R. (1995). Load transfer across the pelvic bone. J. Biomech. 28 (6), 715–724. 10.1016/0021-9290(94)00125-n 7601870

[B7] de Andrada Pereira B. Lehrman J. N. Sawa A. G. U. Lindsey D. P. Yerby S. A. Godzik J. (2021). Biomechanical effects of a novel posteriorly placed sacroiliac joint fusion device integrated with traditional lumbopelvic long-construct instrumentation. J. Neurosurg. Spine 35, 320–329. 10.3171/2020.11.Spine201540 34144523

[B8] de Andrada Pereira B. Wangsawatwong P. Lehrman J. N. Sawa A. G. U. Lindsey D. P. Yerby S. A. (2022). Biomechanics of a laterally placed sacroiliac joint fusion device supplemental to S2 alar-iliac fixation in a long-segment adult spinal deformity construct: A cadaveric study of stability and strain distribution. J. Neurosurg. Spine 36 (1), 42–52. 10.3171/2021.3.Spine202175 34534964

[B9] Devlin V. J. Boachie-Adjei O. Bradford D. S. Ogilvie J. W. Transfeldt E. E. (1991). Treatment of adult spinal deformity with fusion to the sacrum using CD instrumentation. J. Spinal Disord. 4 (1), 1–14. 1839666

[B10] Dinizo M. Passias P. Kebaish K. Errico T. J. Raman T. (2021). The approach to pseudarthrosis after adult spinal deformity surgery: Is a multiple-rod construct necessary? Glob. Spine J., 21925682211001880. 10.1177/21925682211001880 PMC10240611 33858226

[B11] Eastlack R. K. Soroceanu A. Mundis G. M. Jr. Daniels A. H. Smith J. S. Line B. (2022). Rates of loosening, failure, and revision of iliac fixation in adult deformity surgery. Spine (Phila Pa 1976) 47 (14), 986–994. 10.1097/brs.0000000000004356 35819333

[B12] El Dafrawy M. H. Raad M. Okafor L. Kebaish K. M. (2019). Sacropelvic fixation: A comprehensive review. Spine Deform. 7 (4), 509–516. 10.1016/j.jspd.2018.11.009 31202365

[B13] Elmasry S. Asfour S. Travascio F. (2017). Effectiveness of pedicle screw inclusion at the fracture level in short-segment fixation constructs for the treatment of thoracolumbar burst fractures: A computational biomechanics analysis. Comput Methods Biomech. Biomed. Engin 20 (13), 1412–1420. 10.1080/10255842.2017.1366995 28817960

[B14] Finger T. Bayerl S. Onken J. Czabanka M. Woitzik J. Vajkoczy P. (2014). Sacropelvic fixation versus fusion to the sacrum for spondylodesis in multilevel degenerative spine disease. Eur. Spine J. 23 (5), 1013–1020. 10.1007/s00586-014-3165-6 24448893

[B15] Gao Z. Sun X. Chen C. Teng Z. Xu B. Ma X. (2021). Comparative radiological outcomes and complications of sacral-2-alar iliac screw versus iliac screw for sacropelvic fixation. Eur. Spine J. 30 (8), 2257–2270. 10.1007/s00586-021-06864-7 33987735

[B16] Godzik J. Hlubek R. J. Newcomb A. Lehrman J. N. de Andrada Pereira B. Farber S. H. (2019). Supplemental rods are needed to maximally reduce rod strain across the lumbosacral junction with TLIF but not ALIF in long constructs. Spine J. 19 (6), 1121–1131. 10.1016/j.spinee.2019.01.005 30684758

[B17] Guevara-Villazón F. Boissiere L. Hayashi K. Larrieu D. Ghailane S. Vital J. M. (2020). Multiple-rod constructs in adult spinal deformity surgery for pelvic-fixated long instrumentations: An integral matched cohort analysis. Eur. Spine J. 29 (4), 886–895. 10.1007/s00586-020-06311-z 31993784

[B18] Guler U. O. Cetin E. Yaman O. Pellise F. Casademut A. V. Sabat M. D. (2015). Sacropelvic fixation in adult spinal deformity (ASD); a very high rate of mechanical failure. Eur. Spine J. 24 (5), 1085–1091. 10.1007/s00586-014-3615-1 25323138

[B19] Gupta A. Cha T. Schwab J. Fogel H. Tobert D. G. Razi A. E. (2021). Osteoporosis is under recognized and undertreated in adult spinal deformity patients. J. Spine Surg. 7 (1), 1–7. 10.21037/jss-20-668 33834122 PMC8024750

[B20] Hakim N. S. King A. I. (1979). A three dimensional finite element dynamic response analysis of a vertebra with experimental verification. J. Biomech. 12 (4), 277–292. 10.1016/0021-9290(79)90070-8 468853

[B21] Hallager D. W. Karstensen S. Bukhari N. Gehrchen M. Dahl B. (2017). Radiographic predictors for mechanical failure after adult spinal deformity surgery: A retrospective cohort study in 138 patients. Spine (Phila Pa 1976) 42 (14), E855–e863. 10.1097/brs.0000000000001996 27879571

[B22] Han B. Yin P. Hai Y. Cheng Y. Guan L. Liu Y. (2021). The comparison of spinopelvic parameters, complications, and clinical outcomes after spinal fusion to S1 with or without additional sacropelvic fixation for adult spinal deformity: A systematic review and meta-analysis. Spine (Phila Pa 1976) 46 (17), E945–e953. 10.1097/brs.0000000000004003 34384096

[B23] Han S. Hyun S. J. Kim K. J. Jahng T. A. Lee S. Rhim S. C. (2017). Rod stiffness as a risk factor of proximal junctional kyphosis after adult spinal deformity surgery: Comparative study between cobalt chrome multiple-rod constructs and titanium alloy two-rod constructs. Spine J. 17 (7), 962–968. 10.1016/j.spinee.2017.02.005 28242335

[B24] Hasan M. Y. Liu G. Wong H. K. Tan J. H. (2020). Postoperative complications of S2AI versus iliac screw in spinopelvic fixation: A meta-analysis and recent trends review. Spine J. 20 (6), 964–972. 10.1016/j.spinee.2019.11.014 31830594

[B25] He Z. Zhang M. Li W. Long Z. Wang L. Li Q. Q. (2021). Finite element analysis of an improved correction system for spinal deformity. Vivo 35 (4), 2197–2205. 10.21873/invivo.12491 PMC8286466 34182497

[B26] Hirase T. Shin C. Ling J. Phelps B. Haghshenas V. Saifi C. (2022). S2 alar-iliac screw versus traditional iliac screw for spinopelvic fixation: A systematic review of comparative biomechanical studies. Spine Deform. 10 (6), 1279–1288. 10.1007/s43390-022-00528-2 35763199

[B27] Hsiao C. K. Tsai Y. J. Yen C. Y. Li Y. C. Hsiao H. Y. Tu Y. K. (2022). Biomechanical effect of hybrid dynamic stabilization implant on the segmental motion and intradiscal pressure in human lumbar spine. Bioeng. (Basel) 10 (1), 31. 10.3390/bioengineering10010031 PMC9854656 36671603

[B28] Hyun S. J. Lenke L. G. Kim Y. C. Koester L. A. Blanke K. M. (2014). Comparison of standard 2-rod constructs to multiple-rod constructs for fixation across 3-column spinal osteotomies. Spine 39 (22), 1899–1904. 10.1097/BRS.0000000000000556 25299168

[B29] Jain A. Brooks J. T. Kebaish K. M. Sponseller P. D. (2016). Sacral alar iliac fixation for spine deformity. JBJS Essent. Surg. Tech. 6 (1), e10. 10.2106/jbjs.St.15.00074 30237920 PMC6145611

[B30] Jung J. M. Hyun S. J. Kim K. J. Jahng T. A. (2019). Rod fracture after multiple-rod constructs for adult spinal deformity. J. Neurosurg. Spine 32, 407–414. 10.3171/2019.9.Spine19913 31783347

[B31] Kawahara N. Murakami H. Yoshida A. Sakamoto J. Oda J. Tomita K. (2003). Reconstruction after total sacrectomy using a new instrumentation technique: A biomechanical comparison. Spine (Phila Pa 1976) 28 (14), 1567–1572. 10.1097/01.brs.0000076914.32408.85 12865846

[B32] Kelly B. P. Shen F. H. Schwab J. S. Arlet V. Diangelo D. J. (2008). Biomechanical testing of a novel four-rod technique for lumbo-pelvic reconstruction. Spine (Phila Pa 1976) 33 (13), E400–E406. 10.1097/BRS.0b013e31817615c5 18520925

[B33] Kiapour A. Joukar A. Elgafy H. Erbulut D. U. Agarwal A. K. Goel V. K. (2020). Biomechanics of the sacroiliac joint: Anatomy, function, biomechanics, sexual dimorphism, and causes of pain. Int. J. Spine Surg. 14 (1), 3–13. 10.14444/6077 32123652 PMC7041664

[B34] Kim H. J. Yang J. H. Chang D. G. Suk S. I. Suh S. W. Song K. S. (2020). Adult spinal deformity: Current concepts and decision-making strategies for management. Asian Spine J. 14 (6), 886–897. 10.31616/asj.2020.0568 33254357 PMC7788366

[B35] Kim J. H. Horton W. Hamasaki T. Freedman B. Whitesides T. E. Jr. Hutton W. C. (2010). Spinal instrumentation for sacral-pelvic fixation: A biomechanical comparison between constructs ending with either S2 bicortical, bitriangulated screws or iliac screws. J. Spinal Disord. Tech. 23 (8), 506–512. 10.1097/BSD.0b013e3181c37438 20124912

[B36] Kim Y. J. Bridwell K. H. Lenke L. G. Cho K. J. Edwards C. C. 2nd (2006a). Pseudarthrosis in adult spinal deformity following multisegmental instrumentation and arthrodesis. J. Bone Jt. Surg. Am. 88 (4), 721–728. 10.2106/jbjs.E.00550 16595461

[B37] Kim Y. J. Bridwell K. H. Lenke L. G. Rhim S. Cheh G. (2006b). Pseudarthrosis in long adult spinal deformity instrumentation and fusion to the sacrum: Prevalence and risk factor analysis of 144 cases. Spine (Phila Pa 1976) 31 (20), 2329–2336. 10.1097/01.brs.0000238968.82799.d9 16985461

[B38] Lamas V. Charles Y. P. Tuzin N. Steib J. P. (2021). Comparison of degenerative lumbar scoliosis correction and risk for mechanical failure using posterior 2-rod instrumentation versus 4-rod instrumentation and interbody fusion. Eur. Spine J. 30 (7), 1965–1977. 10.1007/s00586-021-06870-9 33993350

[B39] Lau D. Haddad A. F. Fury M. T. Deviren V. Ames C. P. (2021). Multilevel pedicle subtraction osteotomy for correction of severe rigid adult spinal deformities: A case series, indications, considerations, and literature review. Oper. Neurosurg. Hagerst. 20 (4), 343–354. 10.1093/ons/opaa419 33377144

[B40] Lee K. Y. Lee J. H. Kang K. C. Shin S. J. Shin W. J. Im S. K. (2020). Strategy for obtaining solid fusion at L5-S1 in adult spinal deformity: Risk factor analysis for nonunion at L5-S1. J. Neurosurg. Spine 33, 323–331. 10.3171/2020.2.Spine191181 32302980

[B41] Lee N. J. Marciano G. Puvanesarajah V. Park P. J. Clifton W. E. Kwan K. (2022a). Incidence, mechanism, and protective strategies for 2-year pelvic fixation failure after adult spinal deformity surgery with a minimum six-level fusion. J. Neurosurg. Spine 38, 208–216. 10.3171/2022.8.Spine22755 36242579

[B42] Lee N. J. Park P. J. Puvanesarajah V. Clifton W. E. Kwan K. Morrissette C. R. (2022b). How common is acute pelvic fixation failure after adult spine surgery? A single-center study of 358 patients. J. Neurosurg. Spine 38, 91–97. 10.3171/2022.7.Spine22498 36029261

[B43] Lertudomphonwanit T. Kelly M. P. Bridwell K. H. Lenke L. G. McAnany S. J. Punyarat P. (2018). Rod fracture in adult spinal deformity surgery fused to the sacrum: Prevalence, risk factors, and impact on health-related quality of life in 526 patients. Spine J. 18 (9), 1612–1624. 10.1016/j.spinee.2018.02.008 29501749

[B44] Leszczynski A. Meyer F. Charles Y. P. Deck C. Bourdet N. Willinger R. (2022). Influence of double rods and interbody cages on range of motion and rod stress after spinopelvic instrumentation: A finite element study. Eur. Spine J. 31 (6), 1515–1524. 10.1007/s00586-022-07149-3 35461384

[B45] Lindsey D. P. Perez-Orribo L. Rodriguez-Martinez N. Reyes P. M. Newcomb A. Cable A. (2014). Evaluation of a minimally invasive procedure for sacroiliac joint fusion - an *in vitro* biomechanical analysis of initial and cycled properties. Med. Devices (Auckl) 7, 131–137. 10.2147/mder.S63499 24868175 PMC4031207

[B46] Lyu Q. Lau D. Haddad A. F. Deviren V. Ames C. P. (2022). Multiple-rod constructs and use of bone morphogenetic protein–2 in relation to lower rod fracture rates in 141 patients with adult spinal deformity who underwent lumbar pedicle subtraction osteotomy. J. Neurosurg. Spine 36 (2), 1–11. 10.3171/2021.3.SPINE201968 34560633

[B47] Martin C. T. Polly D. W. Holton K. J. San Miguel-Ruiz J. E. Albersheim M. Lender P. (2022). Acute failure of S2-alar-iliac screw pelvic fixation in adult spinal deformity: Novel failure mechanism, case series, and review of the literature. J. Neurosurg. Spine 36 (1), 53–61. 10.3171/2021.2.Spine201921 34479206

[B48] Merrill R. K. Kim J. S. Leven D. M. Kim J. H. Cho S. K. (2017). Multi-rod constructs can prevent rod breakage and pseudarthrosis at the lumbosacral junction in adult spinal deformity. Glob. Spine J. 7 (6), 514–520. 10.1177/2192568217699392 PMC5582710 28894680

[B49] Ntilikina Y. Charles Y. P. Persohn S. Skalli W. (2020). Influence of double rods and interbody cages on quasistatic range of motion of the spine after lumbopelvic instrumentation. Eur. Spine J. 29 (12), 2980–2989. 10.1007/s00586-020-06594-2 32936405

[B50] Oe S. Narita K. Hasegawa K. Natarajan R. N. Yamato Y. Hasegawa T. (2021). Longer screws can reduce the stress on the upper instrumented vertebra with long spinal fusion surgery: A finite element analysis study. Glob. Spine J. 21925682211018467, 21925682211018467. 10.1177/21925682211018467 PMC10189329 34002639

[B51] Palumbo M. A. Shah K. N. Eberson C. P. Hart R. A. Daniels A. H. (2015). Outrigger rod technique for supplemental support of posterior spinal arthrodesis. Spine J. 15 (6), 1409–1414. 10.1016/j.spinee.2015.03.004 25771756

[B52] Panjabi M. M. Oxland T. R. Yamamoto I. Crisco J. J. (1994). Mechanical behavior of the human lumbar and lumbosacral spine as shown by three-dimensional load-displacement curves. J. Bone Jt. Surg. Am. 76 (3), 413–424. 10.2106/00004623-199403000-00012 8126047

[B53] Park S. J. Park J. S. Lee C. S. Lee K. H. (2021a). Metal failure and nonunion at L5-S1 after long instrumented fusion distal to pelvis for adult spinal deformity: Anterior versus transforaminal interbody fusion. J. Orthop. Surg. Hong. Kong) 29 (3), 230949902110542. 10.1177/23094990211054223 34874195

[B54] Park S. J. Park J. S. Nam Y. Yum T. H. Choi Y. T. Lee C. S. (2021b). Failure types and related factors of spinopelvic fixation after long construct fusion for adult spinal deformity. Neurosurgery 88 (3), 603–611. 10.1093/neuros/nyaa469 33372223

[B55] Pellisé F. Vila-Casademunt A. Ferrer M. Domingo-Sàbat M. Bagó J. Pérez-Grueso F. J. (2015). Impact on health related quality of life of adult spinal deformity (ASD) compared with other chronic conditions. Eur. Spine J. 24 (1), 3–11. 10.1007/s00586-014-3542-1 25218732

[B56] Phillips A. T. Pankaj P. Howie C. R. Usmani A. S. Simpson A. H. (2007). Finite element modelling of the pelvis: Inclusion of muscular and ligamentous boundary conditions. Med. Eng. Phys. 29 (7), 739–748. 10.1016/j.medengphy.2006.08.010 17035063

[B57] Rabinovich E. P. Buell T. J. Wang T. R. Shaffrey C. I. Smith J. S. (2021). Reduced occurrence of primary rod fracture after adult spinal deformity surgery with accessory supplemental rods: Retrospective analysis of 114 patients with minimum 2-year follow-up. J. Neurosurg. Spine 35 (4), 504–515. 10.3171/2020.12.Spine201527 34298503

[B58] Ramey W. L. Jack A. S. Chapman J. R. (2021). The lexicon of multirod constructs in adult spinal deformity: A concise description of when, why, and how. J. Neurosurg. Spine 36, 1023–1029. 10.3171/2021.10.Spine21745 34972079

[B59] Rohlmann A. Bauer L. Zander T. Bergmann G. Wilke H. J. (2006). Determination of trunk muscle forces for flexion and extension by using a validated finite element model of the lumbar spine and measured *in vivo* data. J. Biomech. 39 (6), 981–989. 10.1016/j.jbiomech.2005.02.019 16549091

[B60] Safaee M. M. Ames C. P. Smith J. S. (2020). Epidemiology and socioeconomic trends in adult spinal deformity care. Neurosurgery 87 (1), 25–32. 10.1093/neuros/nyz454 31620794

[B61] Sardi J. P. Lazaro B. Smith J. S. Kelly M. P. Dial B. Hills J. (2022). Rod fractures in thoracolumbar fusions to the sacrum/pelvis for adult symptomatic lumbar scoliosis: Long-term follow-up of a prospective, multicenter cohort of 160 patients. J. Neurosurg. Spine 38, 217–229. 10.3171/2022.8.Spine22423 36461845 PMC10193478

[B62] Sayed D. Amirdelfan K. Naidu R. K. Raji O. R. Falowski S. (2021). A cadaver-based biomechanical evaluation of a novel posterior approach to sacroiliac joint fusion: Analysis of the fixation and center of the instantaneous Axis of rotation. Med. Devices (Auckl) 14, 435–444. 10.2147/mder.S347763 34949942 PMC8691588

[B63] Schmidt H. Heuer F. Drumm J. Klezl Z. Claes L. Wilke H. J. (2007). Application of a calibration method provides more realistic results for a finite element model of a lumbar spinal segment. Clin. Biomech. (Bristol, Avon) 22 (4), 377–384. 10.1016/j.clinbiomech.2006.11.008 17204355

[B64] Shen F. H. Qureshi R. Tyger R. Lehman R. Singla A. Shimer A. (2018). Use of the "dual construct" for the management of complex spinal reconstructions. Spine J. 18 (3), 482–490. 10.1016/j.spinee.2017.08.235 28887273

[B65] Shi B. Liu D. Zhu Z. Wang Y. Li Y. Liu Z. (2021). Sequential correction technique in degenerative scoliosis with type C coronal imbalance: A comparison with traditional 2-rod technique. J. Neurosurg. Spine 36, 1005–1011. 10.3171/2021.10.Spine21768 34952513

[B66] Shi D. Wang F. Wang D. Li X. Wang Q. (2014). 3-D finite element analysis of the influence of synovial condition in sacroiliac joint on the load transmission in human pelvic system. Med. Eng. Phys. 36 (6), 745–753. 10.1016/j.medengphy.2014.01.002 24508529

[B67] Shirazi-Adl S. A. Shrivastava S. C. Ahmed A. M. (1984). Stress analysis of the lumbar disc-body unit in compression. A three-dimensional nonlinear finite element study. Spine (Phila Pa 1976) 9 (2), 120–134. 10.1097/00007632-198403000-00003 6233710

[B68] Sohn S. Park T. H. Chung C. K. Kim Y. J. Jang J. W. Han I. B. (2018). Biomechanical characterization of three iliac screw fixation techniques: A finite element study. J. Clin. Neurosci. 52, 109–114. 10.1016/j.jocn.2018.03.002 29580745

[B69] Son D. M. Lee S. B. Lee S. J. Park T. H. Jang J. E. Jeong S. J. (2022). Biomechanical comparison of multilevel lumbar instrumented fusions in adult spinal deformity according to the upper and lower fusion levels: A finite element analysis. Biomed. Res. Int. 2022, 1–9. 10.1155/2022/2534350 PMC9729043 36506913

[B70] Tang Z. Hu Z. Zhu Z. Qiao J. Mao S. Ling C. (2022). The utilization of dual second sacral alar-iliac screws for spinopelvic fixation in patients with severe kyphoscoliosis. Orthop. Surg. 14 (7), 1457–1468. 10.1111/os.13348 35698273 PMC9251291

[B71] Uotani K. Tanaka M. Sonawane S. Ruparel S. Fujiwara Y. Arataki S. (2021). Comparative study of bilateral dual sacral-alar-iliac screws versus bilateral single sacral-alar-iliac screw for adult spine deformities. World Neurosurg. 156, e300–e306. 10.1016/j.wneu.2021.09.048 34560299

[B72] Wang T. Liu H. Zheng Z. Li Z. Wang J. Shrivastava S. S. (2013). Biomechanical effect of 4-rod technique on lumbosacral fixation: An *in vitro* human cadaveric investigation. Spine (Phila Pa 1976) 38(15), E925–E929. 10.1097/BRS.0b013e3182967968 23609200

[B73] Yamanaka K. Mori M. Yamazaki K. Kumagai R. Doita M. Chiba A. (2015). Analysis of the fracture mechanism of Ti-6Al-4V alloy rods that failed clinically after spinal instrumentation surgery. Spine (Phila Pa 1976) 40 (13), E767–E773. 10.1097/brs.0000000000000881 25785960

[B74] Yamato Y. Hasegawa T. Togawa D. Yoshida G. Banno T. Arima H. (2020). Long additional rod constructs can reduce the incidence of rod fractures following 3-column osteotomy with pelvic fixation in short term. Spine Deform. 8 (3), 481–490. 10.1007/s43390-020-00071-y 32072487

[B75] Yang H. Liu J. Hai Y. Han B. (2023). What are the benefits of lateral lumbar interbody fusion on the treatment of adult spinal deformity: A systematic review and meta-analysis deformity. Glob. Spine J. 13 (1), 172–187. 10.1177/21925682221089876 PMC9837508 35442824

[B76] Yang H. Liu J. Hai Y. (2021). Is instrumented lateral lumbar interbody fusion superior to stand-alone lateral lumbar interbody fusion for the treatment of lumbar degenerative disease? A meta-analysis. J. Clin. Neurosci. 92, 136–146. 10.1016/j.jocn.2021.08.002 34509241

[B77] Zheng N. Watson L. G. Yong-Hing K. (1997). Biomechanical modelling of the human sacroiliac joint. Med. Biol. Eng. Comput 35 (2), 77–82. 10.1007/bf02534134 9136197

