# Supplementary material for: Biomechanical evaluation of multiple pelvic screws and multirod construct for the augmentation of lumbosacral junction in long spinal fusion surgery
Source: Front Bioeng Biotechnol. 2023 Mar 14;11:1148342. doi: 10.3389/fbioe.2023.1148342 (PMC10043192; doi:10.3389/fbioe.2023.1148342)
Supplement: Supplementary file 1 [file DataSheet1.PDF]

Supplementary Material

Biomechanical evaluation of multiple pelvic screws and multirod construct for the augmentation of lumbosacral junction in long spinal fusion surgery

Honghao Yang*, Aixing Pan, Yong Hai, Fengqi Cheng, Hongtao Ding, and Yuzeng Liu

*** Correspondence:** Yong Hai: yong.hai@ccmu.edu.cn

1. Supplementary Figures

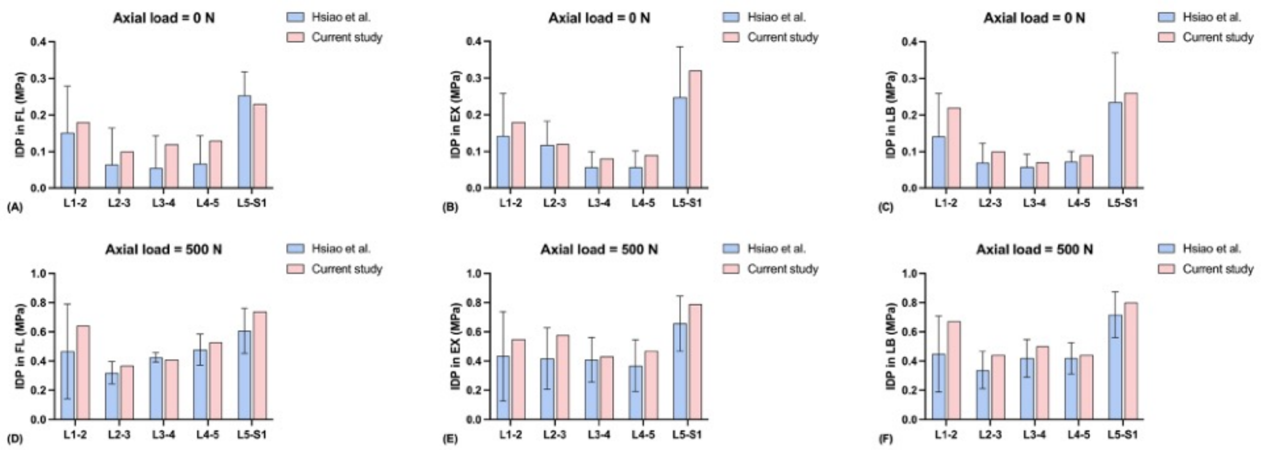

Supplementary Figure S1. Comparison of the intradiscal pressure between the intact model and in-vitro study.

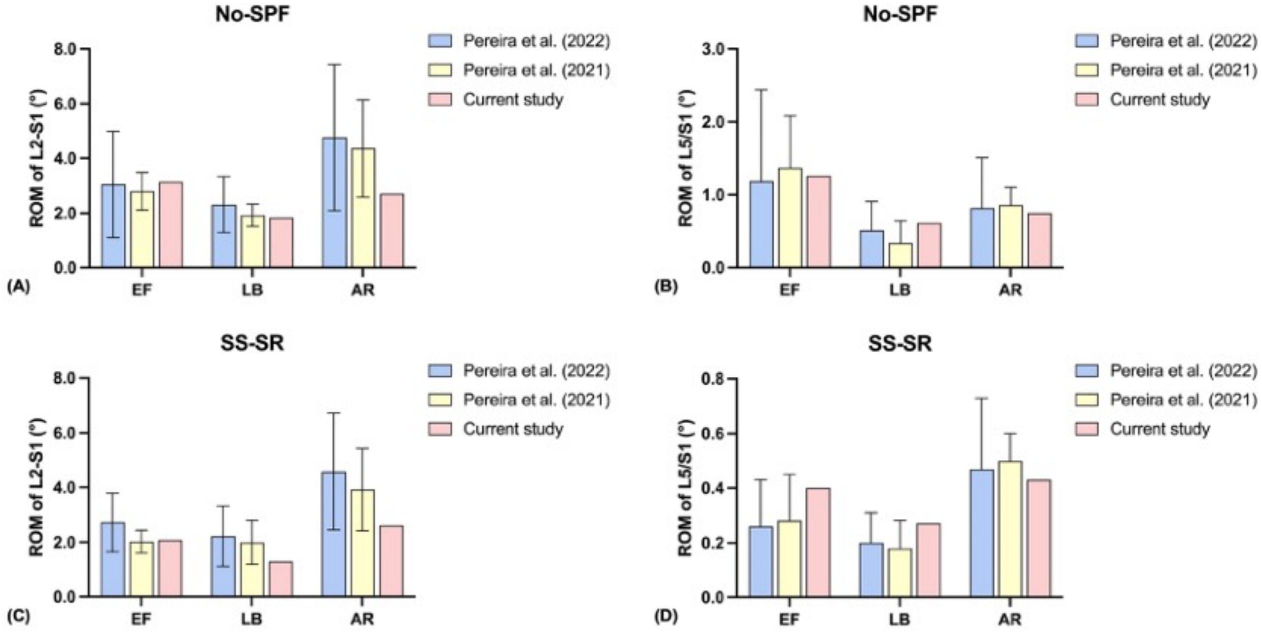

Supplementary Figure S2**.** Comparison of the range of motion between the instrumented models and in-vitro studies.

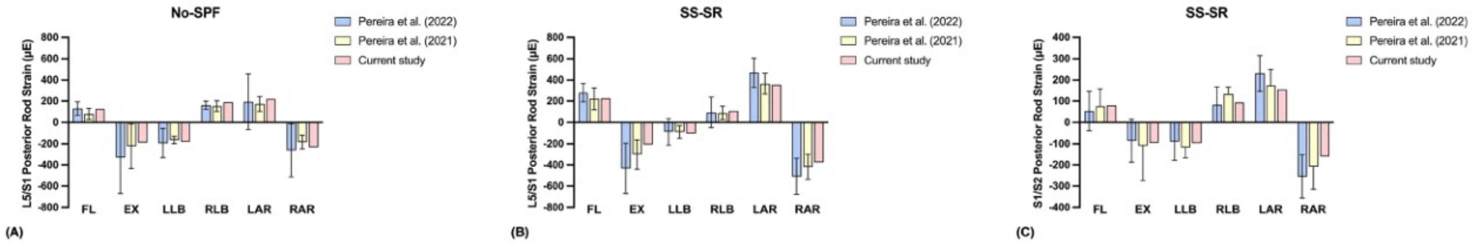

Supplementary Figure S3. Comparison of the rod strains between the instrumented models and in-vitro studies.

2. Supplementary Tables

Supplementary Table S1. Range of motion of the intact model and instrumented models

ROM (°)	Model	FL	EX	LB	AR
**Global**	**Intact**	29.810	17.502	41.460	18.510
	**No-SPF**	2.548	1.582	2.240	2.241
	**SS-SR**	1.708	0.961	1.536	1.384
	**MS-SR**	1.250	0.628	1.233	1.056
	**SS-MR**	1.202	0.672	1.467	1.084
	**MS-MR**	0.964	0.440	1.117	0.944
**LSJ**	**Intact**	7.634	4.406	6.657	2.649
	**No-SPF**	1.259	0.731	1.155	1.078
	**SS-SR**	0.934	0.456	0.776	0.688
	**MS-SR**	0.684	0.256	0.608	0.519
	**SS-MR**	0.542	0.300	0.597	0.447
	**MS-MR**	0.418	0.230	0.440	0.340
**SIJ**	**Intact**	1.642	0.791	1.050	1.632
	**No-SPF**	1.675	0.807	1.064	1.644
	**SS-SR**	0.301	0.215	0.470	0.640

**MS-SR**	0.096	0.081	0.290	0.153
**SS-MR**	0.284	0.231	0.472	0.611
**MS-MR**	0.103	0.073	0.310	0.149

ROM, range of motion; LSJ, lumbosacral junction; SIJ, sacroiliac joint; FL, flexion; EX, extension; LB, lateral bending; AR, axial rotation

Supplementary Table S2. von-Mises stress on the intact model and instrumented models

VMS (MPa)	Model	FL	EX	LB	AR
**Instrumentation**	**No-SPF**	156.00	108.5	139.00	174.40
	**SS-SR**	145.40	92.30	125.50	157.32
	**MS-SR**	139.30	82.10	118.70	148.74
	**SS-MR**	128.60	83.70	107.00	153.26
	**MS-MR**	125.40	67.10	102.90	141.87
**Lumbosacral rods**	**No-SPF**	102.92	108.5	124.21	140.84
	**SS-SR**	98.50	92.35	119.12	137.00
	**MS-SR**	96.24	86.73	117.64	132.90
	**SS-MR**	88.79	72.63	105.64	125.10
	**MS-MR**	80.23	59.24	99.41	102.99
**S1-PS**	**No-SPF**	70.74	48.31	55.21	72.28

	**SS-SR**	56.53	40.55	50.25	60.74
	**MS-SR**	43.60	27.79	45.08	51.69
	**SS-MR**	37.84	29.61	41.66	54.01
	**MS-MR**	31.39	21.48	35.44	43.30
**Cages**	**No-SPF**	67.32	33.56	38.76	51.94
	**SS-SR**	54.46	30.88	39.32	45.57
	**MS-SR**	46.10	25.53	39.00	43.63
	**SS-MR**	53.82	25.80	31.02	40.94
	**MS-MR**	42.26	23.16	28.78	38.06
**S1-SEP**	**No-SPF**	49.05	25.66	48.85	37.25
	**SS-SR**	44.33	23.12	42.66	32.34
	**MS-SR**	34.52	18.66	43.26	30.03
	**SS-MR**	42.29	17.61	35.32	26.65
	**MS-MR**	33.06	15.53	34.77	23.47
**Sacrum**	**No-SPF**	96.45	45.55	54.36	79.01
	**SS-SR**	60.86	41.28	46.77	66.71
	**MS-SR**	50.18	33.65	42.32	52.83
	**SS-MR**	61.74	39.25	42.34	54.56

**MS-MR**	36.00	32.48	36.25	43.33

PS, pedicle screw; SEP, superior endplate; FL, flexion; EX, extension; LB, lateral bending; AR, axial rotation
